# Reaction kinetics of anodic biofilms under changing substrate concentrations: Uncovering shifts in Nernst‐Monod curves via substrate pulses

**DOI:** 10.1002/elsc.202100088

**Published:** 2022-01-19

**Authors:** Fabian Kubannek, Jonathan Block, Balakrishnan Munirathinam, Rainer Krull

**Affiliations:** ^1^ Institute of Energy and Process Systems Engineering Technische Universität Braunschweig Braunschweig Germany; ^2^ Institute of Biochemical Engineering Technische Universität Braunschweig Braunschweig Germany; ^3^ Center of Pharmaceutical Engineering (PVZ) Technische Universität Braunschweig Braunschweig Germany

**Keywords:** anodic biofilms, bioelectrochemical system, concentration dependency, Nernst‐Monod model, reaction kinetics

## Abstract

In the present study, it is shown that the concentration dependency of undefined mixed culture anodic biofilms does not follow a single kinetic curve, such as the Nernst‐Monod curve. The biofilms adapt to concentration changes, which inevitably have to be applied to record kinetic curves, resulting in strong shifts of the kinetic parameters. The substrate concentration in a continuously operated bioelectrochemical system was changed rapidly via acetate pulses to record Nernst‐Monod curves which are not influenced by biofilm adaptation processes. The values of the maximum current density *j*_max_ and apparent half‐saturation rate constant *K*_s_ increased from 0.5 to 1 mA cm^−2^ and from 0.5 to 1.6 mmol L^−1^, respectively, within approximately 5 h. Double pulse experiments with a starvation phase between the two acetate pulses showed that *j*_max_ and *K*_s_ decrease reversibly through an adaptation process when no acetate is available. Pseudo‐capacitive charge values estimated from non‐turnover cyclic voltammograms (CV) led to the hypothesis that biofilm adaptation and the observed shift of the Nernst‐Monod curves occurred due to changes in the concentration of active redox proteins in the biofilm. It is argued that concentration‐related parameters of kinetic models for electroactive biofilms are only valid for the operating points where they have been determined and should always be reported with those conditions.

**Abbreviations**
BES: bioelectrochemical system
CA: chronoamperometry
CV: cyclic voltammogram
HPLC: high performance liquid chromatography

## 1 INTRODUCTION

For the design of efficient, reliable, industrial‐scale bioelectrical systems (BES), profound knowledge of the reaction kinetics, the substrate consumption and the electrochemical performances are of utmost importance. A key aspect of performance optimization in BES lies in the knowledge and comprehensive understanding of the underlying reaction kinetics. Furthermore, the kinetic expression are an essential part of most BES models [1, 2, 3, 4, 5]. Unfortunately, the reaction kinetics are not well established in the bioelectrochemical literature. This is partly due to a lack of standardized methods for investigating kinetics and due to the large variety of defined and undefined electroactive mixed cultures being tested under vastly differing cultivation conditions.

Finding suitable and flexible reaction kinetic models for the simultaneously occurring biological and electrochemical processes in BES is an ongoing challenge till now. One approach to describe the reaction kinetics is the Nernst‐Monod model incorporating the anodic potential dependency of the specific substrate uptake rate *q*_S_ (mmol_S_ g_CDW_^−1^ h^−1^) in BES [4]. Since substrate consumption in BES is usually accompanied by current generation, *q_S_* is often described through the current density *j* (mA cm^−2^).

(1) $$\begin{array}{c} \begin{array}{c} j=j_{\max}\cdot X_{\mathrm{bf}}\cdot \left(\frac{c_{S}}{K_{S}+c_{S}}\right)\cdot \left(\frac{1}{1+\exp\left[-\frac{F}{\mathrm{RT}}\cdot \left(E_{A}-E_{\mathrm{KA}}\right)\right]}\right) \end{array} \end{array}$$

with the maximum current density *j*_max_, the active biofilm‐associated biomass *X*_bf_, the growth‐limiting substrate concentration *c_S_*, the potential difference *(E*_A_ *‐E*_KA_) between the anode potential *E*_A_ and the potential *E*_KA_ at which *j *= 1/2 *j*_max_, the Faraday constant *F*, the universal gas constant *R*, the cultivation temperature *T* and the apparent half‐saturation rate constant *K*_S_ of *X*_bf_. Here, the term “*apparent*” is used because it refers to the overall *K*_S_ of the electrode‐associated biofilm and not to the Monod constant which reflects the relationship between planktonic bacterial growth and the growth limiting substrate.

**PRACTICAL APPLICATION**

Bioelectrochemical systems (BES) are an emerging sustainable technology which employs electroactive microorganisms for applications ranging from wastewater treatment to the production of value‐added products from various feedstocks. Under anaerobic cultivation conditions, electroactive microorganisms can degrade dissolved organic compounds and generate electrical current by using a solid electrode as electron acceptor. Municipal wastewater treatment is regarded as one of the main fields of BES applications because both electricity generation as well as the degradation of organic compounds are desirable goals in wastewater treatment. However, sudden changes in dissolved substrate availability and overall wastewater composition occur frequently because of intra‐day fluctuations or when surface water from rain runs into the sewer and dilutes the organic compounds. Thus, a comprehensive understanding of reaction kinetics and adaptation processes in electroactive biofilms in response to changing process conditions is essential to enhance the environmental performance, economic viability and to model and simulate BES processes to generate suitable data for a scale up.

Also, other model equations have been proposed, such as the Butler‐Volmer‐Monod model which combines the previously described Nernst‐Monod model with the Butler‐Volmer model for electron transfer kinetics. According to Hamelers et al. [6], the combined model was better at predicting apparent half‐saturation rate constants *K*_S_ at varying electrode potentials in kinetic experiments.

The focus of these models is placed on the effect of the electrode potential. The influence from the substrate concentration is studied far less than the influence of electrode potential. One reason might be fact that the potential dependency is a defining and rare feature of electroactive microorganisms. However, for design and operation of BES, understanding the effect of substrate concentration on kinetics is equally important.

In experiments, the reaction kinetic parameters of *j*_max_ and *K*_S_ of kinetic models are usually determined by changing the growth‐limiting concentration *c*_s_ in a continuous BES system and simultaneously measuring the resulting current generation. The correlation of two variables forms the shape of the underlying Nernst‐Monod curve.

However, the main challenge for the experimental determination of the kinetic parameters is the fact that adaptation processes such as biomass growth, or biomass lysis take place in parallel to current production. Thus, the kinetic parameters may change during the experiments to record them. Previous studies indicated that biomass growth and adaptation occur on the same time scale as substrate concentration changes for BES batch reactors with common lab‐scale electrode sizes and reactor volumes [7, 8]. This makes it difficult to record kinetic curves in such reactors by concentration sampling during the substrate depletion phase. In literature, Nernst‐Monod curves are mostly recorded using biofilms that were grown at high substrate concentrations in continuous flow reactors. The substrate concentration is changed and each concentration value is applied for multiple days to record the kinetic curves [4, 6] so that adaptation of the biofilm to the changing conditions can be expected.

**FIGURE 1:**
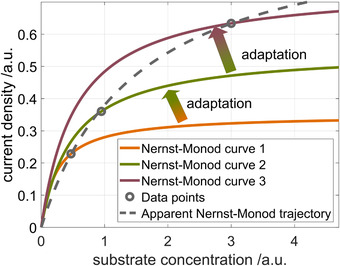
Schematic illustration of different Nernst‐Monod curves which result from microbial adaptation processes of a single electroactive biofilm, and an apparent Nernst‐Monod trajectory which may be constructed from experimental steady state data points

This circumstance leads to the fact that, when several successive measurements are carried out, data points will be obtained which appear to follow an apparent Nernst‐Monod curve, but do not reflect the behavior of the biofilm in a defined state. This shifting from one trajectory to another on an apparent Nernst‐Monod curve due to growth is exemplified in Figure 1 for increasing substrate concentrations. Initially the biofilm which was grown at a low *c*_S_ can be described by Nernst‐Monod curve 1. When the concentration is increased, an adaptation process is triggered which results in the new Nernst‐Monod curve 2. After a second concentration increase, curve 3 is reached. When only a number of discrete data points are taken into account, an apparent Nernst‐Monod relationship may be constructed which does not reflect any of the underlying kinetic curves.

In this work, two research goals are targeted: The first goal is to develop an approach to obtain true Nernst‐Monod relationships for the concentration dependency of anodic biofilms without the influence of adaptation processes. It should be noted that these “*true*” Nernst‐Monod relationships for biofilms are not necessarily identical to Monod‐curves in planktonic culture. The same applied for the values of the kinetic parameters. The second goal is to investigate the adaptation process shown in Figure 1 which causes significant shifts in the Nernst‐Monod relationship upon slow changes in substrate conditions.

In a “classical” chemostat process, the growth‐limiting (chemical) substrate is continuously added and removed and is kept at a constant *c*_S_ in the feed flow. Equal influent and effluent flow rates are maintained to retain a constant working volume. As a result, the substrate concentration and biomass concentration are kept at a steady state (d*c*_S_/d*t* = d*c*_CDW_/d*t* = 0) with a constant specific growth rate *μ* and metabolic activity. This makes a chemostat an ideal experimental setup to study the metabolic performances and physiology of microbial systems. One of the most important features of the chemostat is that it allows the operator to control *μ* by adjusting the averaged hydraulic retention time. For a BES, the same approaches apply, except that in the biomass balance the free, planktonic biomass concentration is extended by the term of the immobilized biomass concentration, which is retained by cell immobilization on an electrode resulting in the accumulation of biomass [2, 3]. If the BES is operated continuously, this corresponds to a retentostat process. Under these conditions it is very challenging to obtain a steady state of the biomass (d*c*_CDW_/d*t *= 0) because, after changing the concentration, the biofilms may continue growing or changing their structure resulting in the behavior illustrated in Figure 1. To measure true Nernst‐Monod trajectories in such a BES retentostat without or with low interference of the microbial growth, rapid changes in substrate concentration can be applied. Applying substrate concentration pulses by injecting substrate into a retentostat could make the growth influence negligible, especially when the disturbance of the substrate pulse would quickly be nullified by the continuous operation under substrate‐limiting conditions. Until now, substrate pulses were used to investigate metabolic pathways and interaction in BES [9, 10, 11] yielding promising results.

However, substrate pulses have not been widely used to determine reaction kinetics for describing the concentration response behavior of BES. Therefore, in this work, acetate pulse injections are applied to a continuously operated acetate‐fed mixed culture BES retentostat to obtain Nernst‐Monod curves and to investigate adaptation phenomena of the biofilms as a response to changes in substrate availability.

## 2 MATERIALS AND METHODS

### 2.1 Setup

**FIGURE 2:**
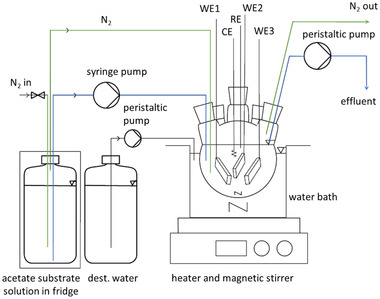
Schematic drawing of the experimental setup. Blue lines indicate acetate substrate solution, green lines nitrogen. WE1‐3 are the three working electrodes, RE is the reference electrode, CE is the counter electrode

A schematic drawing of the setup is depicted in Figure 2. The experiments were performed in a round bottom reactor (working liquid volume 250 mL) consisting of a 5‐necked glass flask which was placed inside a water bath at 35°C and agitated by a magnetic stirrer (diameter 12 mm, 150 min^−1^). An undefined mixed culture biofilm (inoculum see chapter 2.2) was grown on three identical rectangular graphite anodes (50 mm x 10 mm x 5 mm) which served as working electrodes. The top side of the electrodes was covered with epoxy resin resulting in an accessible surface area of 15.5 cm^2^ per electrode. A platinum wire was used as a counter electrode. The reference electrode was a saturated Ag/AgCl electrode (SE11, Xylem Analytics, Germany). The reactor was supplied with acetate as solely carbon source and continuously flushed with 20 mL min^−1^ nitrogen (99.999%) to avoid oxygen intrusion and the accumulation of hydrogen from the counter electrode. The reactor was operated in half‐cell mode under potentiostatic control of a potentiostat (Gamry Instruments 1010E, USA). Chronoamperometry (CA) was recorded at a constant potential of 0.2 V, cyclic voltammograms (CV) were recorded from 0.2 to ‐0.5 V at a scan rate of 1 mV s^−1^. All potentials are reported with respect to a saturated Ag/AgCl reference electrode (+0.197 V vs. SHE). At 0.2 V the current generation is not limited by the electrode potential and the potential dependent term of the Nernst‐Monod equation approaches one. Thus, the effect of *c*_S_ can be studied without interfering effects or limitations resulting from the electrode potential.

During continuous flow operation, acetate substrate solution was pumped into the reactor by a double syringe pump (Nemesys, Cetoni GmbH, Germany) equipped with two 10 mL glass syringes. A peristaltic pump was used to pump the effluent out of the reactor and keep the liquid volume constant. If not noted otherwise, the flow rate was 0.4 mL min^−1^, corresponding to an averaged hydraulic retention time of 10.4 h and a dilution rate of 0.096 h^−1^, respectively. To prevent microbial growth and acetate consumption in the storage container, substrate solution was stored in a fridge and replaced at latest every 4 days. Additionally, the substrate solution container was continuously bubbled with nitrogen. High performance liquid chromatography (HPLC) analysis confirmed that no acetate consumption occurred in the substrate solution container.

To investigate the adaptation of the anodic biofilms to changes in substrate availability, acetate pulses of 2 mL with 410 mmol L^−1^ were applied when the reactor was in steady state (numerical criterion for steady state: |d*c*_S_/d*t*| < 0.0015 mmol L^−1^ h^−1^ and |d*j*/d*t*| <  0.0017 mA cm^−2^ h^−1^). The concentrated acetate solution was injected directly into the reactor using a syringe. After a brief mixing time, a 2 mL sample for HPLC analysis was taken directly out of the reactor to avoid volume changes.

The three anodes were extracted from the reactor one by one at different times of cultivation for the determination of the cell dry weight density (*CDWD*) related to the electrode area. At the same time the feed solution container was exchanged, and the acetate concentration was adjusted so that the steady state concentration in the reactor remained constant despite of the lower consumption after extraction of one anode. For each anode that was extracted, a new bare anode was inserted into the reactor to avoid changes in the liquid volume. This newly inserted anode was not connected electrically so that no bacteria would grow on it that could falsify the current signal by oxidizing acetate electrochemically.

### 2.2 Inoculum and media composition

Substrate solution was produced by dissolving sodium acetate in a pH 7 buffer solution containing 2.69 g L^−1^ NaH_2_PO_4_, 4.33 g L^−1^ Na_2_HPO_4_, 0.31 g L^−1^ NH_4_Cl, 0.31 g L^−1^ KCl [12], 12.5 mL L^−1^ trace element mixture, and 12.5 mL L^−1^ vitamin mixture. Following Balch et al. [13], the vitamin solution contained 2 mg L^−1^ biotin, 2 mg L^−1^ folic acid, 10 mg L^−1^ pyridoxine hydrochloride, 5 mg L^−1^ thiamine hydrochloride, 5 mg L^−1^ riboflavin, 5 mg L^−1^ nicotinic acid, 5 mg L^−1^ DL‐calcium pantothenate, 100 μg L^−1^ vitamin B_12_, 5 mg L^−1^ *p*‐aminobenzoic acid and 5 mg L^−1^ lipoic acid. The trace element solution contained 1.5 g L^−1^ nitrilotriacetic acid, 3 g L^−1^ MgSO_4_· 7 H_2_O, 500 mg L^−1^ MnSO_4_· 2 H_2_O, 1 g L^−1^ NaCl, 100 mg L^−1^ FeSO_4_· 7 H_2_O, 100 mg L^−1^ CoCl_2_, 100 mg L^−1^ CaCl_2_· 2 H_2_O, 130 mg L^−1^ ZnSO_4_, 100 mg L^−1^ CuSO_4_ ·H_2_O, 100 mg L^−1^ AlK(SO_4_)_2_, 100 mg L^−1^ H_3_BO_3_, 10 mg L^−1^ Na_2_MoO_4_· 2 H_2_O, 300 μg L^−1^ Na_2_SeO_3_· 5 H_2_O and 30 mg L^−1^ NiCl_2_· 6 H_2_O. The solution was deaerated with nitrogen (99.999%) for 20 min before use.

The acetate concentration in the substrate feed solution was 4 mmol L^−1^ in the beginning, 3 mmol L^−1^ after the first anode had been extracted, and 2.1 mmol L^−1^ after two anodes had been extracted. Pre‐tests performed in our lab (data not shown) had shown that this adjustment of the acetate concentration in the feed holds the current density and acetate concentration in the reactor constant upon removing the anodes. In the end of an experiment the reactor was operated under a continuous supply of substrate solution without acetate to record data points at low acetate concentrations. In some experiments, these conditions were also applied before the end of the experiment. In the latter case, these operating points will be referred to as starvation conditions.

The inoculum originated from a mixed‐culture biofilm dominated by *Geobacter sulfurreducens*/*anodireducens* enriched from municipal wastewater (wastewater treatment plant Steinhof, Braunschweig, Germany). The enrichment was performed as described by Riedl et al. [14]. Before starting an experiment, the reactor was filled with 250 mL of a deaerated substrate solution containing 4 mmol L^−1^ sodium acetate, vitamins, and trace minerals. Bacteria were scraped off an anode directly into the solution. After inoculation, the reactor was kept in batch mode for 2 days before the continuous flow operation was started to allow the bacteria to immobilize on the electrodes.

### 2.3 Biomass quantification and concentration measurements

Acetate concentrations were determined by HPLC (Dionex UltiMate 3000, Thermo Fischer Scientific, USA), with a diode array detector (DAD‐3000(RS), Thermo Fischer Scientific, USA) at 210 nm. For analysis, a Brownlee Validated Aqueous C18 column (No. N9303549, Perkin Elmer) was applied using H_3_PO_4_ at pH 2.4 and acetonitrile 80/20 (v/v) (flow rate 1.5 mL min^−1^) as eluent with an acetate retention time of approximately 2.1 min. The column and the detector were operated at 25°C. Samples of 2 mL in volume were collected regularly from the effluent at the end of the reactor outlet and filtered through a 0.2 μm pore size regenerated cellulose syringe filter to remove bacteria and particles. No other pretreatment was performed.

The *CDWD* of the biofilm related to the electrode area was determined by removing an anode from the reactor and scraping off the entire biofilm into a centrifuge tube filled with water. The tube was centrifuged at 1666 g for 20 min. The supernatant was discarded, and the pellet was dried overnight at 80°C before determining the *CDWD* gravimetrically. Since all three electrodes are subject to the same conditions, it is assumed that the biofilm on the three electrodes develops in a similar manner. This way the development of biomass over time can be assessed by analyzing the *CDWD* of the three anodes that were taken out of the reactor at different times of cultivation. To confirm that comparable biofilms developed on all three electrodes, the relative contribution of the individual electrodes to the total current were measured regularly, and control experiments were performed in which all three anodes were extracted at the same time to check if the *CDWD* on all electrodes was equal. Since literature examining the microbial composition of anodic biofilms shows that *Geobacter sulfurreducens* becomes the dominant species in many BES cultivations, experiments examining the microbial composition on our anodes were not performed in this work [15, 16, 17].

## 3 RESULTS AND DISCUSSION

### 3.1 Determining Nernst‐Monod curves from substrate pulse responses

In this section, the determination of the true Nernst‐Monod curves from acetate pulse responses will be shown. It will be demonstrated how these curves change within a short cultivation time as a result of adaptation processes in the biofilm.

**FIGURE 3:**
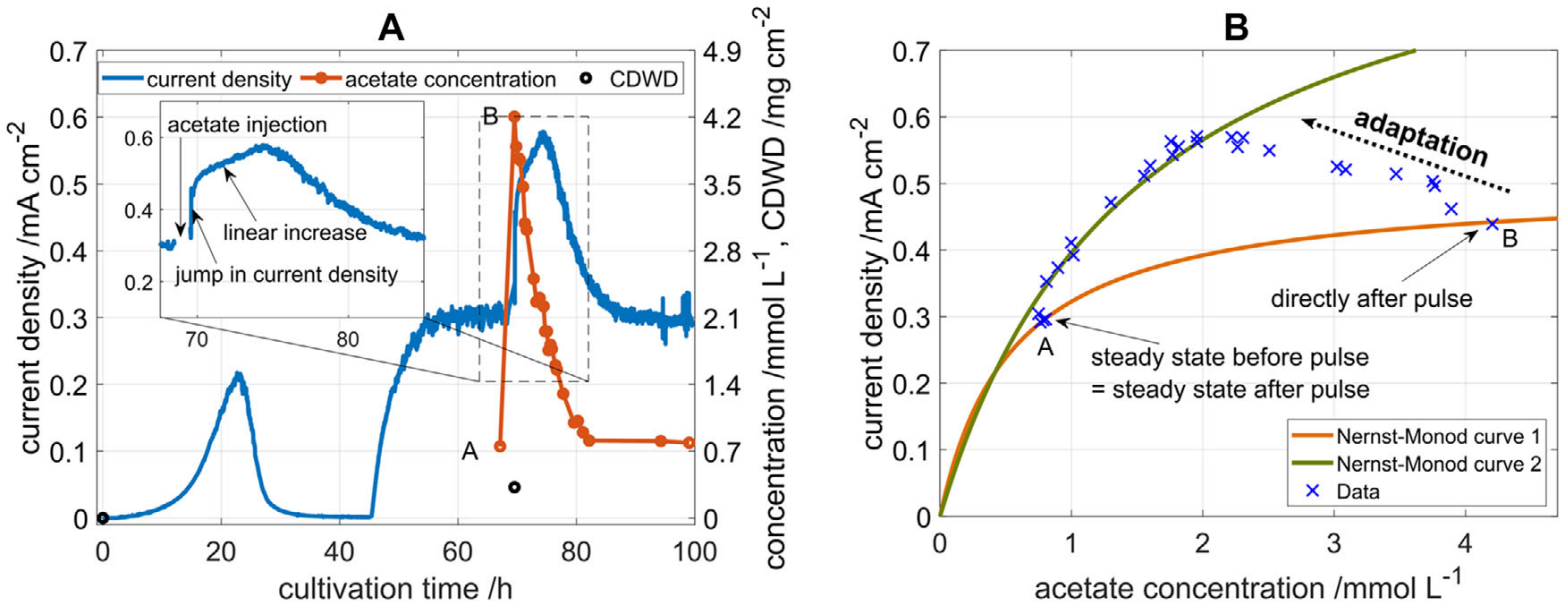
(A) Current density, acetate concentration, and *CDWD* over cultivation time during an acetate pulse experiment. Continuous flow operation was started at 45 h, flow rate is 0.4 mL min^−1^, acetate feed concentration is 4 mmol L^−1^, reactor volume is 0.25 L. (B) Nernst‐Monod plot for the acetate pulse experiment. The measured data points and the Nernst‐Monod curves before (1, orange, short term response) and after (2, green, long term response) the pulse are shown

In Figure 3A, the development of current density and acetate concentration of a continuously operated BES reactor is shown during initial batch phase, constant operation, and an acetate pulse. After the initial batch phase, the continuous flow operation was started at 45 h of cultivation. At 60 h, the current density stabilized at a level of 0.3 mA cm^−^^2^. In the following, an operating point at which the acetate concentration and current density are stable (|d*c*_S_/d*t*| < 0.0015 mmol L^−1^ h^−1^ and |d*j*/d*t*| < 0.0017 mA cm^−2^ h^−1^) is referred to as steady state, although changes in the biofilm structure can still occur.

At 68 h, an acetate pulse was applied to the reactor by injecting a concentrated acetate solution. At the same time, the first one of the three anodes was extracted from the reactor for *CDWD* determination and replaced by a pristine graphite electrode that was not connected electrically. Furthermore, the acetate feed concentration was adjusted to 3 mmol L^−1^ to compensate for the lower acetate consumption. The acetate concentration rapidly increased from the steady state value of 0.7 to 4.2 mmol L^−1^. Subsequently, the current increased to 0.44 mA cm^−^^2^ within 4 min. This time period is attributed to mixing in the liquid phase, diffusion into the biofilm and transport through the cell membranes. Thus, the jump in current density by 0.14 mA cm^−^^2^ or 46 % (c.f. the insert in Figure 3A) reflects the true response of the biofilm to an increase in substrate and is not influenced by adaptation processes. Subsequently, a linear increase in current density was observed, which continued for 4 h until a current density of 0.57 mA cm^−^^2^ was reached. Concomitantly, the acetate concentration was continuously dropping because of increased acetate consumption and the washout due to the continuous flow operation. This result clearly indicates that the linear increase in current density is solely caused by an adaptation of the bacteria in the biofilm. Moreover, the increase in current density does not follow an exponential increase, which would be expected for a growing microbial culture, and can be observed in the batch phase of the BES. The nature of the adaptation process as well as the biomass determined from the extracted anode will be discussed in detail below. After the pulse, current density and acetate concentration returned to their steady state values from before the acetate pulse.

In Figure 3B, the current density is plotted over the measured acetate concentration to examine the Nernst‐Monod trajectory of the BES during the acetate pulse from Figure 3A. The two kinetic parameters, *j*_max_ and *K*_S_, describing the concentration term of the Nernst‐Monod model can be determined algebraically from two data points. The data point directly after the pulse is not yet influenced by any adaptation process. Thus, it can be assumed that the biofilm is in the original state, and the two highlighted data points directly before and directly after the acetate pulse lie on the Nernst‐Monod curve 1 for the biofilm in steady state. This is the Nernst‐Monod curve for the biofilm without the influence of adaptation. The following data pairs represent the adaptation phase, in which the acetate concentration drops whereas the current density increases. Once the acetate concentration reaches a value of 2 mmol L^−1^ the adaptation phase is over, and both current density and acetate concentration decrease following a different Nernst‐Monod curve 2. This data directly illustrates how a biofilm can adjust its substrate response behavior as described by Nernst‐Monod curves within a few hours. The velocity and the extent of the adaptation indicate that such adaptation processes would occur in most experiments to record bioelectrochemical kinetics like Nernst‐Monod [18], Butler‐Volmer‐Monod [6], or other kinetic behavior [19] in mixed‐culture BES.

**FIGURE 4:**
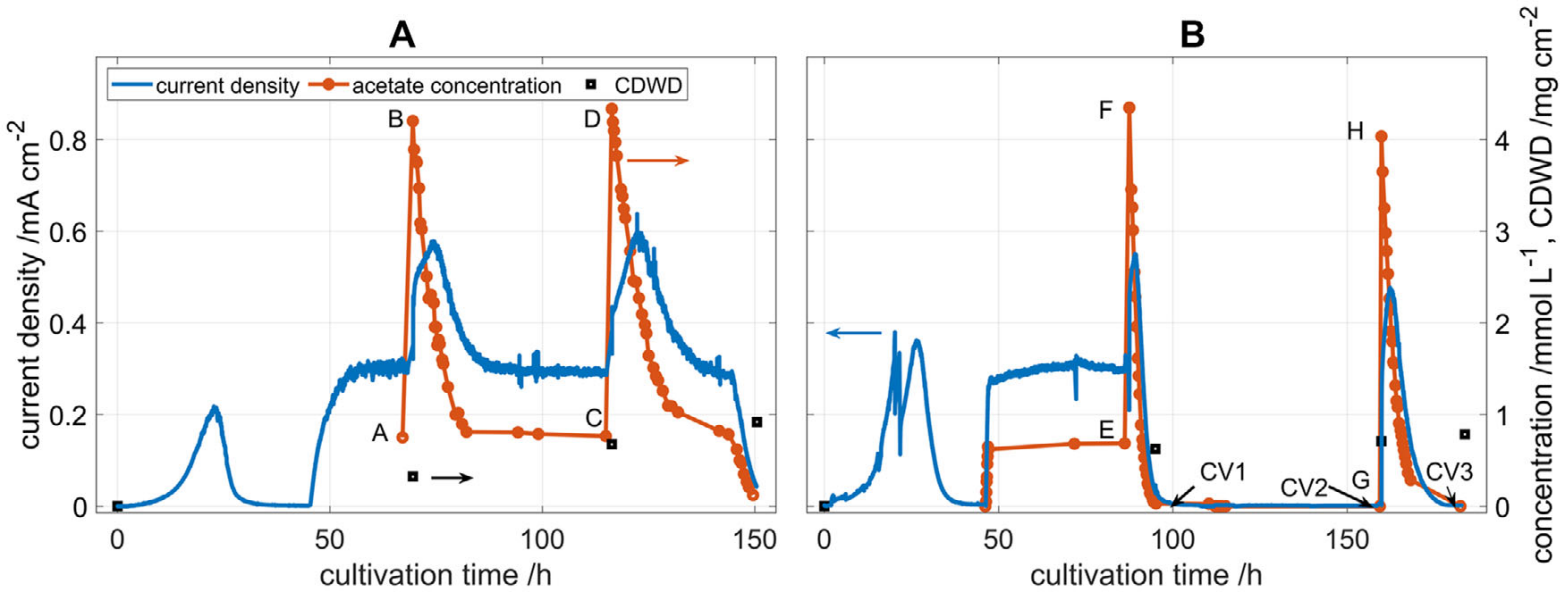
Current density, acetate concentration, and *CDWD* over cultivation time during two acetate pulses with (A) continuous acetate supply between two acetate pulses, and (B) starvation phase without acetate supply between two acetate pulses. Continuous flow operation was started at 45 h, flow rate is 0.4 mL min^−1^, acetate feed concentration is 4 mmol L^−1^, reactor volume is 0.25 L. The letters A‐H relate the data points to the Nernst‐Monod curves in Figure 5. CV1 to CV3 are related to CV discussed in Figure 7

To investigate the nature of the adaptation progress, and to ascertain if the adaptation of the biofilm was perpetual, a second acetate pulse was applied 2 days after the first acetate pulse. This continuation of the experiment from Figure 3A is shown in Figure 4A. The reactor was continuously supplied with substrate between the two acetate pulses. The current density and acetate concentration returned to their steady‐state values after the acetate pulses, and the response to the second pulse closely resembled the response to the first pulse. Small changes in the steady state current density directly might result from an increase in biofilm thickness or a change in the ratio of living to dead cells. The variations in current density in the start‐up phase between Figure 4A and B result from changes in the activity of the inoculum. The acetate concentration dropped more slowly after the second pulse because a second anode was extracted for biomass determination before the second pulse. This lowers the total acetate consumption rate.

In Figure 4B, results from a different experiment are displayed. The experimental procedure was left unchanged, only the feed concentration of acetate was set to zero after the first acetate pulse so that no carbon source was supplied anymore. Thus, the biofilm is operated under starvation conditions after the first acetate pulse. Current density and acetate concentration dropped to zero and remained there until the second acetate pulse was applied. After the second acetate pulse, the current density reached lower values than after the first pulse. The CV recorded at the points marked by CV1 to CV3 in Figure 4B will be discussed later on.

**FIGURE 5:**
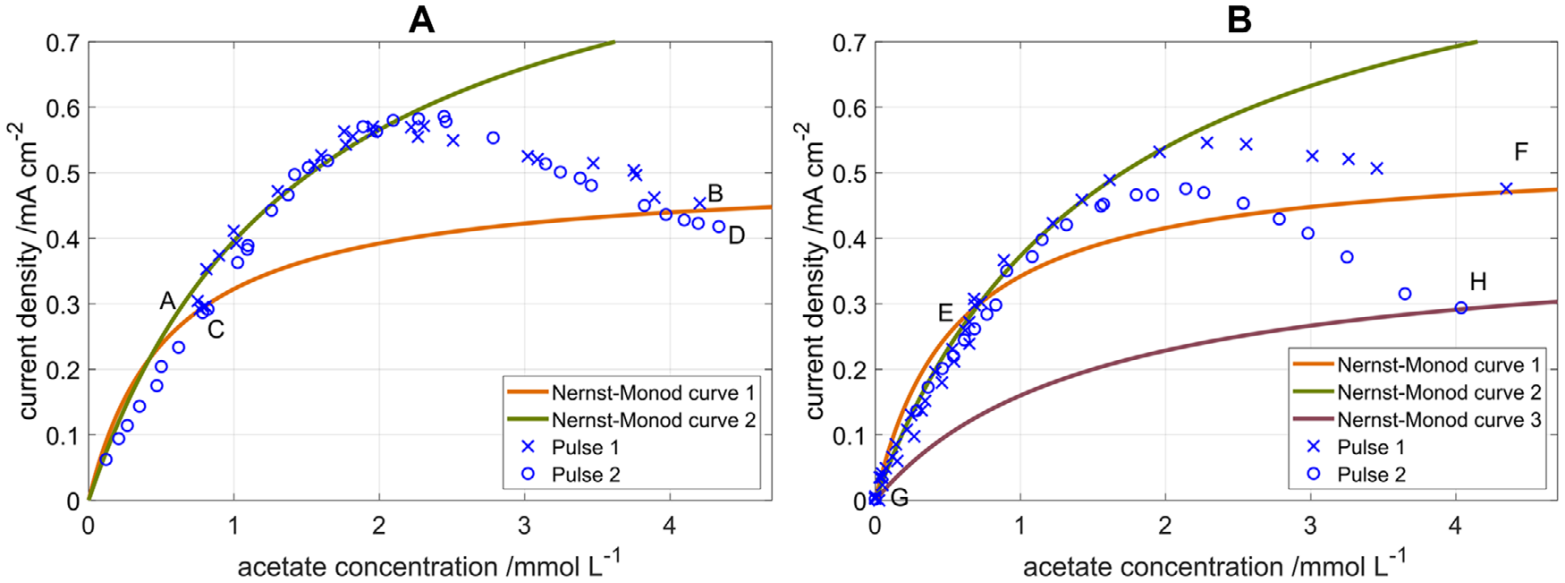
Nernst‐Monod plot for two consecutive acetate pulses with (A) continuous acetate supply between two acetate pulses (at A and C) and (B) a starvation phase without acetate supply between the two acetate pulses (at F and H). The measured data points are displayed as markers, the solid lines indicate Nernst‐Monod curves that were fitted to the data at different points in cultivation time. Nernst‐Monod curves before (curve 1, orange) and after (curve 2, green) the acetate pulses as well as after the starvation phase (curve 3, purple) are shown. The letters A–H relate the data points to the data over cultivation time from Figure 4

In Figure 5, the Nernst‐Monod curves for the two pulse experiments are shown. In Figure 5A it can be seen that the Nernst‐Monod curves recorded after the two acetate pulses are identical. The steady state conditions before the acetate pulse, indicated by the letters A and C in Figures 4A and 5A, the immediate responses to an acetate pulse, indicated by the letters B and D, as well as the transient marking the adaptation from the Nernst‐Monod curve 1 (orange) to curve 2 (green) are coinciding. This indicates that the biofilm has returned to its original state after two days of operation under steady state conditions. Here the term “*original state*” refers to the current production and its response to changes in acetate concentration. The continuous increase in *CDWD* (compare to Figure 4A and **B**) indicates that the biofilm is still changing and will be discussed below.

In Figure 5B, the Nernst‐Monod curves for the consecutive double pulse experiment under starvation conditions are shown. After the first acetate pulse adaptation takes place shifting the Nernst‐Monod curve 1 (orange) to curve 2 (green). This adaptation process is slightly less pronounced than in Figure 5A because no acetate is supplied anymore at the inlet so that washout and substrate consumption cause the acetate concentration to fall faster. After the second acetate pulse, the immediate responses of the current density, indicated by the letter H in Figures 4B and 5B, is much lower than the response to the first acetate pulse, indicated by letter F. During the starvation phase, the biofilm adapted to the new operating conditions and shifted to the Nernst‐Monod curve 3 (purple). This Nernst‐Monod curve 3 mainly serves as a guide to the eye because an infinite set of parameters can connect the data points G and H. The two experiments with consecutive pulses show that the adaptation process is reversible and works in positive and negative direction. When the continuous reactor is operated at steady state regarding substrate supply and current density after the pulse, the acetate response behavior returns to the steady state behavior; when the biofilm is cultivated under starvation conditions after the acetate pulse, the current density curve in the second pulse is lower.

In Table 1 the reaction kinetic parameters *j*_max_ and *K*_S_ for the Nernst‐Monod curves 1 (before the adaptation process) are shown. In Table 2 the parameters for the Nernst‐Monod curves 2 (after the adaptation process) are shown. The values were determined from four independent experiments, which can be found in Figure 5A and B and Figure S2a and Figure S2b in the supplementary information.

**TABLE 1:** Reaction kinetic parameters of *j*_max_ and *K*_S_ of the Nernst‐Monod curves 1 (before the adaptation process)

Curve	*j*_max_ /mA cm^−2^	*K*_S_ /mmol L^−1^
Figure 5A) curve 1	0.5	0.55
Figure 5B) curve 1	0.53	0.55
Figure S2a) curve 1	0.54	0.58
Figure S2b) curve 1	0.6	0.88
Mean/standard deviation	0.54/0.04	0.64/0.14

**TABLE 2:** Reaction kinetic parameters of *j*_max_ and *K*_S_ of the Nernst‐Monod curves 2 (after the adaptation process)

Curve	*j*_max_/mA cm^−2^	*K*_S_ /mmol L^−1^
Figure 5A) curve 2	0.99	1.5
Figure 5B) curve 2	0.97	1.6
Figure S2a) curve 2	0.89	1.3
Figure S2b) curve 2	1	2
Mean/standard deviation	0.96/0.04	1.6/0.25

The reported half‐saturation rate constants *K*_S_ should be interpreted as apparent half‐saturation rate constants because the acetate concentration in the biofilm can differ from the bulk concentration and exhibit local gradients. Previous reports provided excellent insights on oxygen microelectrode studies of auto‐/heterotrophic biofilms with O_2_ as growth limiting substrate [20, 21, 22, 23]. These investigations show changes of substrate concentration and mass transfer effects in aerobic cultivated biofilms. To the best of the authors' knowledge, there is unfortunately no such robust method of investigation available for bioelectrochemical biofilms with acetate as growth limiting substrate.

The values of *j*_max_ and *K*_S_ increase roughly by a factor of 2 during the adaptation that follows the acetate pulse. The mean value of *j*_max_ for curves 2 of 0.96 mA cm^−2^ agrees well with the upper limit of the current density of approximately 1 mA cm^−2^ that is often observed for undefined mixed culture biofilms on graphite. In curve 3 (Figure 5B), that is, after the starvation phase, a significant reduction in the maximum current density value can be observed. *K*_s_ = 1 mmol L^−1^ and *j*_max_ = 0.43 mA cm^−2^ were used to draw the Nernst‐Monod curve 3. But as explained above, these values cannot be determined unambiguously and should only be interpreted qualitatively. The data clearly show that the biofilms do not follow a single Nernst‐Monod curve under all operation conditions but adapt to changes in acetate concentration within hours resulting in a significant shift on the Nernst‐Monod curves.

The adaptation processes are also evident for apparent *K*_S_ assuming that generated redox proteins interact directly with acetate. The apparent half‐saturation rate constant *K*_S_ describes the apparent substrate affinity of the organism in the biofilm. Low values correspond to a high apparent substrate affinity. Before the acetate pulse the mean *K*_S_ = 0.64 mmol L^−1^ indicates a high apparent affinity of the mixed biofilm consortium towards acetate. After the pulse the mean *K*_S_ increased to 1.6 mmol L^−1^ which could indicate a poorer substrate apparent affinity or limitations through limited biofilm conductivity or pH shifts [24, 25]. At the same time, *j*_max_ is higher in this case than before the acetate pulse. This indicates that no inhibition of electricity generation occurred.

For a BES Hamelers et al. [6] reported *K*_S_ values for acetate as the growth‐limiting substrate of 0.37 and 2.2 mmol L^−1^ with the latter value obtained at conditions that are closer to this reactor. Other papers stated similar values of *K_S_* between 0.59 and 2.86 mmol L^−1^ for acetate‐fed anodic biofilms in continuously operated single‐chamber reactors, but only Kretzschmar et al. worked at similar anode potentials [26]. Lee et al. worked at ‐0.4 V versus Ag/AgCl whereas Zarabadi et al. did not specify the applied anode potential during biofilm growth [27, 28]. In literature, Monod constant values of non‐electroactive planktonically cultivated *Escherichia coli* and *Saccharomyces cerevisiae* vary over more than 2 and 3 orders of magnitude, and it should be stressed that the case of *E. coli* and *S. cerevisiae* does not stand alone [29]. *K*_S_ depends on the respective substrate, the concentration range and the chemostat conditions. Thus, the estimated *K*_S_ values for the present retentostat BES with acetate as growth‐limiting carbon source under substrate pulse and starvation conditions appear to be in a trusted range. Repetition experiments (Figure S2 and Tables 1 and 2) show that these parameters are reproducible when using the same operation conditions and inoculum. In addition, it should be noted that in single‐chamber reactors crossfeeding of cathodically produced H_2_ can occur from cathode to anode [30]. The biomass fraction of H_2_‐degrading methanogens in the biofilm may thus not be negligible in closed of setups. Since the reactor setup was actively purged with nitrogen during the experiment, any influence of the biomass fraction of H_2_‐degrading methanogens in the biofilm is most likely neglectable.

### 3.2 Mechanisms of the biofilm adaptation

In this section, the mechanisms that causes the adaptation and the shift of the Nernst‐Monod curves will be discussed. To get further insights into the adaptation mechanism, each BES reactor was equipped with three anodes which were extracted for *CDWD* determination at three different points in time: Before each substrate pulse and at the end of the experiment. The obtained values of the *CDWD* in mg cm^−2^ of the anode are also plotted in Figure 4. In all experiments the *CDWD* continuously increases from the planktonic inoculum with no *CDWD* in the beginning of the experiment until the end of the experiment. A maximum *CDWD* of 1.1 mg cm^2^ is reached. In no experiment a correlation was observed between the *CDWD* and the current density or the parameters of the Nernst‐Monod curves. The results indicate that *CDWD* is not a decisive factor influencing the concentration response behavior for mixed‐culture biofilms with *CDWD* up to 1.1 mg cm^−2^ as obtained after one to two weeks of cultivation time. This includes positive as well as negative effects and is in accordance with results from Zhou et al. [31] and Sun et al. [32] who observed limitations of the current density only after numerous batch cycles when thick biofilms had developed. Such limitations can result from concentration gradients that develop in the biofilms [4]. It can also be concluded that acetate transport through the biofilm is not limiting under the investigated operating conditions because transport limitations would increase with biofilm thickness.

**FIGURE 6:**
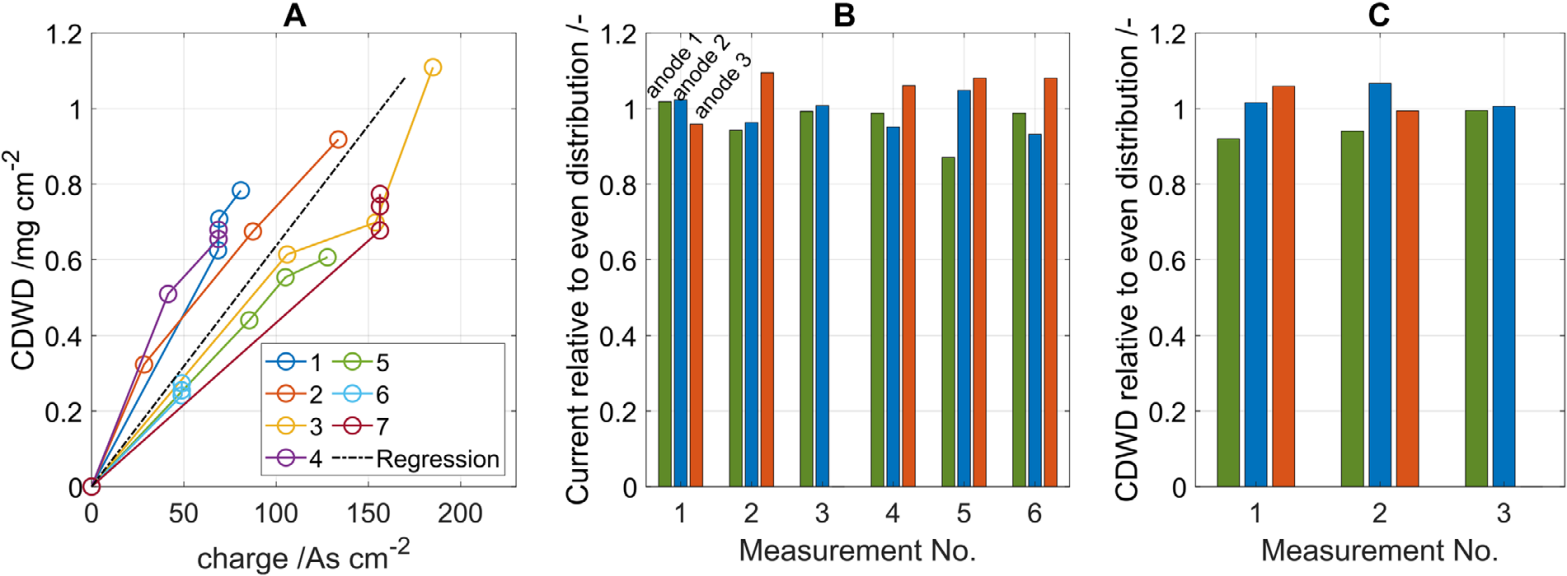
(A) *CDWD* over total charge before anode extraction for seven experiments. To assess the deviations between the electrodes, two anodes were extracted at the same time in experiment 4 and all anodes were extracted at the same time in experiments 6 and 7. (B) Current density delivered by the individual anodes relative to an even distribution measured before extraction of an anode. Measurement No. 1 is from the experiment in Figure 4B, No. 2 and 3 from the experiment in Figure 4A. Measurement No. 3 was done after one anode had already been extracted. Measurements No. 4–6 are from a set control experiments. (C) *CDWD* of anodes from the control experiments which were extracted at the same time relative to an even distribution of the *CDWD*

An approximately linear relation was found between *CDWD* and the total charge that had been transferred by the respective anode up to its extraction (Figure 6A). Thus, *CDWD* was accumulating with electrical current ‐ but not with cultivation time ‐ and had no significant influence on the biofilm behavior. The linear increase with charge results when a constant share of the utilized substrate is directed to the catabolism and biomass degradation is negligible [2]. From a linear regression analysis of the biomass over charge, a yield coefficient of 0.074 mg_CDW_ mg^−1^_acetate_ can be determined after converting the charge to acetate consumption using Faraday's law. Assuming a molar mass of the biomass of 22.4 g mol^−1^ carbon, this corresponds to 9.9% of the carbon from acetate being converted to biomass and is well in the range of literature values [2, 4, 33], for example, Marcus et al. [4] used a value of 10%. Acetate mass balances can be found in Figure S1 in the supplementary information.

The lines in Figure 6A do not show the development of *CDWD* of single anodes. The plot is drawn under the assumption that the *CDWD* of the anodes in the reactor is similar so that the *CDWD* of the anode that has been extracted represents the *CDWD* of the remaining anodes. To check if the anodes exhibit similar characteristics, two tests were performed. The current distribution among the anodes was checked regularly to confirm that the anodes were comparable. In Figure in Figure 6B, the relative distribution of the total current among the anodes directly before extraction of an anode is shown. The values were calculated by dividing the current of each anode by the mean current from all anodes present in the reactor. Some deviations between the anodes exist and are attributed to inhomogeneous flow distribution in the reactor and random fluctuations in the growing biofilm and the changing biofilm surface structure. All in all, the anodes generate comparable current densities. Furthermore, in a set of control experiments, multiple anodes were extracted at the same time and the distribution of the biomass among the anodes was evaluated. The results in Figure 6C show that the amount of biomass on the anodes extracted at the same time is similar. Thus, the biomass from an anode extracted from the reactor at a specific cultivation time allows one to draw conclusions on the biomass on the remaining anodes in the BES reactor.

A second hypothesis that might explain the adaptation of the biofilm and the change in Nernst‐Monod curves is a change in protein expression of the bacteria in response to the change in substrate availability. Here especially redox proteins such as outer membrane cytochromes, which are essential for the transfer of electrons to the electrode, play a vital role. Redox proteins can exchange electrons with the electrode even in the absence of substrate resulting in a pseudo‐capacitive current [34, 35, 36]. In CVs recorded in absence of substrate, so called non‐turnover CVs, these pseudo‐capacitive currents can be measured [36].

**FIGURE 7:**
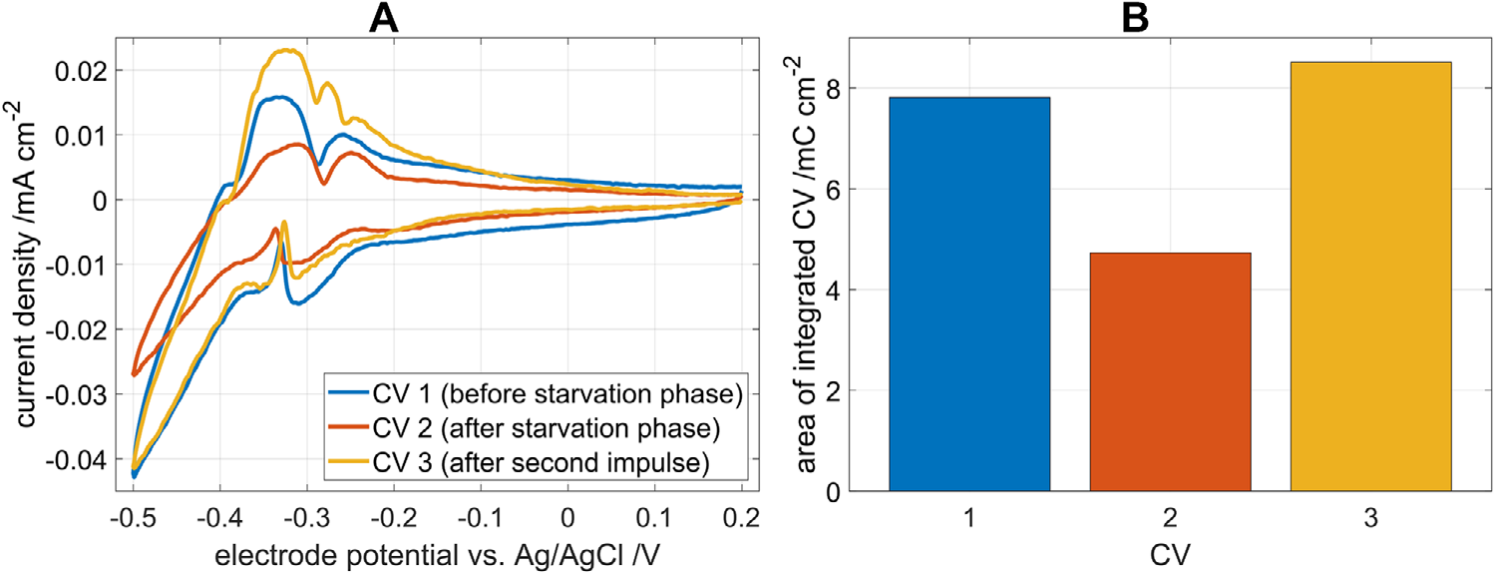
(A) CV under non‐turnover conditions recorded before (CV1), and after the starvation phase (CV2) and after the second acetate pulse (CV3) (compare also to Figure 5B). The CV curves are corrected for the residual (turnover) current. (B) Areas enclosed by the CV obtained by integration

In Figure 7A, three non‐turnover CVs are shown which were recorded during the consecutive double acetate pulse experiment shown in Figure 4B, where the biofilm was starved between the two pulses. Arrows in Figure 4B indicate when the CV1, CV2, and CV3 were recorded; CV1 was recorded after the first acetate pulse, CV2 was recorded after the cell maintenance phase of 2 days, and CV3 was recorded after the second acetate pulse. All CVs were shifted so that the current at 0.2 V is zero to correct for remaining Faradaic (non‐capacitive) current. This is necessary because the oxidation of remaining acetate and extracellular polymeric storage substrates (EPS) in the biofilm can cause current production in the biofilm for a significant amount of time even after substrate supply has been interrupted [35]. No further baseline correction was applied.

In Figure 7B, the values of the area enclosed by the CVs are depicted. These areas reflect the total pseudo‐capacitive charge that is transferred. When electrons are exchanged with the electrode via direct electron transfer as is most likely the case here because the electrode is presumably dominated by *Geobacter* spp., this charge is a function of the amount of active and electrically connected redox proteins [34, 36]. The transferred charge decreases from CV1 to CV2 indicating that such proteins are decomposed during the starvation phase. From CV2 to CV3 the charge increases, indicating that protein synthesis is increased during the second acetate pulse. A direct quantification of the proteins from CVs is challenging because effects such as double layer charging, oxidation of remaining acetate or the oxidation of EPS may interfere even though the CVs were shifted to correct for remaining Faradaic current. Nevertheless, the fact that the charge transferred in CV3 reached similar values as in CV1, agrees well with the with the previously presented results in Figure 5: The Nernst‐Monod curve after the second acetate pulse approaches the Nernst‐Monod curve from the first acetate pulse so that the curves overlay at concentrations below 0.9 mmol L^−1^.

It is not possible to record non‐turnover CVs in the experiments without starvation because their features are only visible in absence of substrate, but it is likely that the same mechanism is valid there. These findings are consistent with findings from Sun et al. [32] as well as Song et al. [37], who showed a correlation between overall protein content and current density of the anode associated biofilm.

Thus, the findings support the second hypothesis which relates shifts in the Nernst‐Mondo curves to changes in the concentration of active electron‐transferring redox proteins in the biofilm. The increase and decrease in maximum current density *j*_max_ observed in Figure 5 may be caused by changes in protein redox concentration.

However, the second hypothesis cannot provide a direct explanation for changes in the apparent *K*_S_ value. It is possible that other factors such as biofilm conductivity [4] or pH‐shifts in the biofilm [38] become limiting at higher current densities which are reached after the adaptation. Such limitations could cause the observed increase of the apparent *K*_S_ value. Finally, further biofilm adaptation processes, which have not been covered here, may cause the change in apparent substrate affinity.

### 3.3 Effects of biofilm history on the Nernst‐Monod curves

In the previous section, it was shown that biofilms which were cultivated under similar conditions exhibit similar Nernst‐Monod curves. When conditions were restored after a disturbance in form of an acetate pulse or a starvation phase, the Nernst‐Monod curves returned to the original curves after some time. In this section, the effect of growing a biofilm at a lower feed concentration of 2.5 mmol L^−1^ is investigated.

**FIGURE 8:**
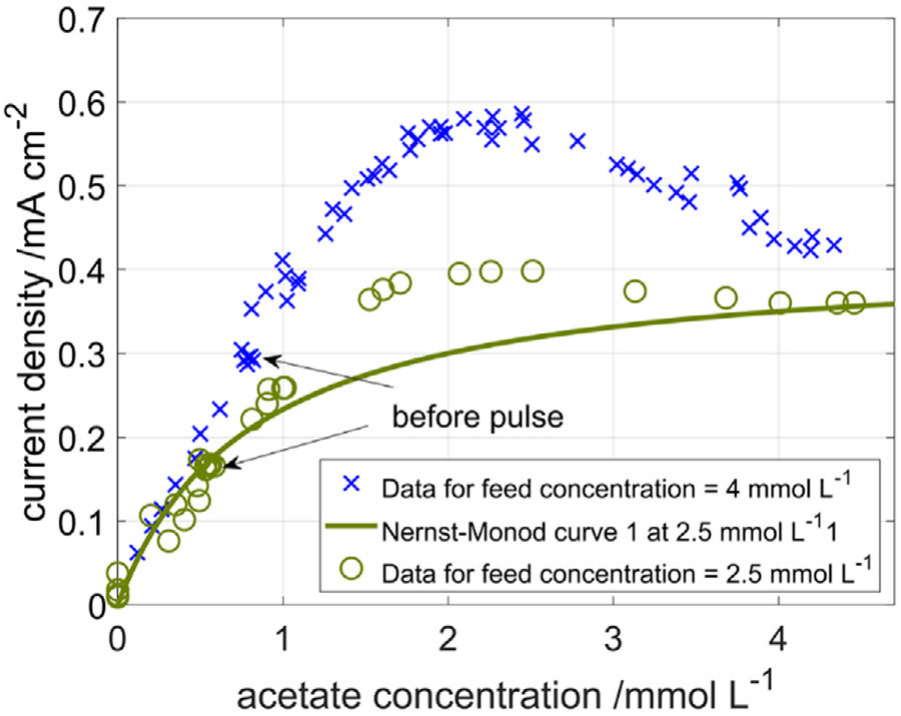
Nernst‐Monod plot for acetate pulse experiments with an acetate feed concentration of 4 (blue crosses) and 2.5 mmol L^−1^ (green circles). Data for 4 mmol L^−1^ is identical to Figure 5A

The results are shown in Figure 8. As expected, the steady state concentration before the acetate pulse as well as the current density is lower at the lower acetate feed concentration of 2.5 mmol L^−1^: 0.5 mmol L^−1^ and 0.18 mA cm^−2^ compared to 0.8 mmol L^−1^ and 0.3 mA cm^−2^, respectively. However, the adaptation rate of the biofilm grown at 2.5 mmol L^−1^ is lower so that the maximum current density does not reach the value of the biofilm grown at 4 mmol L^−1^ before acetate is washed out again. This indicates that factors such as the microbial community or the biofilm structure are shaped by the conditions in the early phase of the continuous operation and continue to determine the adaptation capabilities and the concentration response behavior of the biofilm even under changing conditions. Further research is needed to elucidate which factors are causing this effect.

## 4 CONCLUDING REMARKS

In this study, it was demonstrated that anodic biofilms do not adhere to a single Nernst‐Monod curve but adapt to changes in acetate concentration resulting in strong shifts of their concentration dependency behavior. It is hypothesized that changes in the concentration of active redox proteins play an important role in the adaptation.

In the Nernst‐Monod model [4], *K*_S_ is independent of potential. The Butler‐Volmer‐Monod model [6] extends the Nernst‐Monod model by introducing a dependency of *K*_S_ on the electrode potential. The generated measurements have shown that concentration‐related reaction kinetic parameters also strongly depend on the biofilm history. It was demonstrated that the substrate concentration behavior described by *j*_max_ and *K*_S_ can change significantly within a few hours. Consequently, substrate concentration related terms of kinetic models such as the Nernst‐Monod or Butler‐Volmer‐Monod model are only valid for the operating points where the parameters have been determined. This includes not only the substrate concentration values but also the biofilm history.

The results also have implications for applications of electroactive biofilms in concentration sensors as suggested in the report by Kretzschmar et al. [7]. The effects of adaptation processes to substrate concentration changes may be included in sensor calibration curves, but the results indicate that the time dependency of the adaptation process is a significant factor that has to be taken into account and is not understood very well.

In this work, undefined mixed cultures which performed better than pure cultures regarding power output and general stability [39, 40, 41] were used. In future, the substrate concentration pulse methodology could be transferred to biofilms of defined microbial composition or combined with further structural and electrochemical analysis of the biofilms. This would yield deeper insights into the adaptation mechanisms that allows the biofilm to adjust to substrate concentration changes. Based on such insights, new quantitative models may be established that will enhance the understanding of effects of substrate concentration to a level that has already been reached regarding the effects of electrode potential.

Nevertheless, the apparent Nernst‐Monod curve which is usually recorded in literature (c.f. Figure 1) is highly relevant for practical applications because adaptation happens in technical systems. For practical applications adaptation is generally a desirable process because it allows the biofilm to withstand changing environmental or operating conditions. Still, it can be shown that the biofilm has a distinct substrate concentration behavior for different biofilm states within the window of operation conditions which we investigated. Analyzing this concentration dependency, which can be obtained via the substrate pulse method, will pave the road towards a better understanding of limiting processes in electroactive biofilms.

## NOMENCLATURE

**Table:** 

*c*	[mmol L^–1^]	concentration
*CDW*	[mg]	cell dry weight
*CDWD*	[mg cm^–2^]	cell dry weight density related to the electrode area
*E*	[V]	electrical potential
*F*	[C mol^–1^]	Faraday constant
*j*	[mA cm^–2^]	current density
*K*	[mmol L^–1^]	half‐saturation constant
*q*_S_	[mmol g_CDW_^–1^ h^–1^]	specific substrate uptake rate
*R*	[J mol^–1^ K^–1^]	universal gas constant
*T*	[K]	temperature
*X*	[g]	active biofilm‐associated biomass
Greek symbols		
*μ*	[h^–1^]	specific growth rate
Indices		
A	anode	
bf	biofilm	
KA	anodic potential at which half‐maximum current density is reached	
max	maximum	
S	growth‐limiting substrate	

## CONFLICT OF INTEREST

The authors have declared no conflicts of interest.

## Supporting information

Supporting information

## Data Availability

The data that support the findings of this study are available from the corresponding author upon reasonable request.
