# Supplementary material for: Reaction kinetics of anodic biofilms under changing substrate concentrations: Uncovering shifts in Nernst‐Monod curves via substrate pulses
Source: Eng Life Sci. 2022 Jan 19;22(3-4):152–64. doi: 10.1002/elsc.202100088 (PMC8961052; doi:10.1002/elsc.202100088)
Supplement: Supplementary file 1 — Supporting information [file ELSC-22-152-s001.pdf]

Supplementary Information

Reaction kinetics of anodic biofilms under changing substrate concentrations: Uncovering shifts in Nernst-Monod curves via substrate pulses

Fabian Kubannek[1],

Jonathan Block[2],

Balakrishnan Munirathinam[1],

Rainer Krull[2,3]

[1]Institute of Energy and Process Systems Engineering, Technische Universität Braunschweig, Braunschweig, Germany

[2] Institute of Biochemical Engineering, Technische Universität Braunschweig, Braunschweig, Germany

[3] Center of Pharmaceutical Engineering (PVZ), Technische Universität Braunschweig, Braunschweig, Germany

Correspondence: Dr.-Ing. Fabian Kubannek (f.kubannek@outlook.com). Institute of Energy and Process Systems Engineering, Technische Universität Braunschweig, Langer Kamp 19B, 38106 Braunschweig, Germany

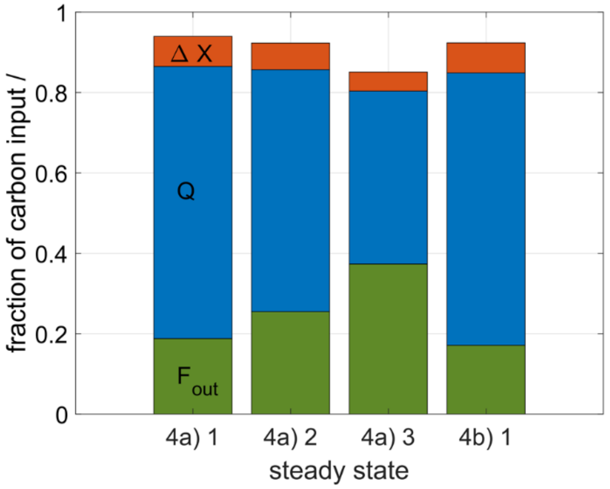

Figure S1: acetate balances for several steady states. (Q = acetate converted to CO_2 by the electrochemical reaction (blue), ΔX = acetate used for to biomass growth determined from the *CDWD* measurements after anode extraction (red), F_{out} = unconsumed acetate leaving the reactor (green)). Steady state 4a) 1 refers to the point in time directly before the first acetate pulse is applied in Figure 4 a) when a (pseudo-) steady state is reached. 4a) 2 refers to the (pseudo-) steady state before the second acetate pulse and 4a), 3 to the (pseudo-) steady state after the second acetate pulse.

In **Figure S1** the acetate balance for the reactor is shown for 4 points from the experiments shown in **Figure 4** in the main text. Steady states 4a) 1 and 4b) 1 represent identical operation conditions and exhibit only minor differences. From steady state 4a) 1 to 4a) 3 the share of unconsumed acetate F_{out} leaving the cell increases continuously, and the share of acetate converted to CO_2 by the electrochemical reaction Q decreases. This results from the extraction of the electrodes which reduces the absolute current while the current density remains constant. The relative error in the acetate balance increases from steady states 4a) 1 to 4a) 3. With decreasing number of anodes present (three anodes in states 4a) 1 and 4b) 1, two anodes in state 4a) 2, and one anode in state 4a) 3), the feed concentration, i.e., the acetate input, is reduced from 4 to 3 to 2.1 mmol L^{-1}. Thus, the increase in the relative error points towards a process that consumes a fixed amount of acetate such as oxygen intrusion and subsequent aerobic acetate oxidation. Additionally, carbon is needed for cell maintenance. Methanogenesis seems unlikely because methanogens grow slowly so that they would be washed at the average hydraulic residence time of 10.4 h. Overall, the balances add up well compared with other mixed culture experiments.

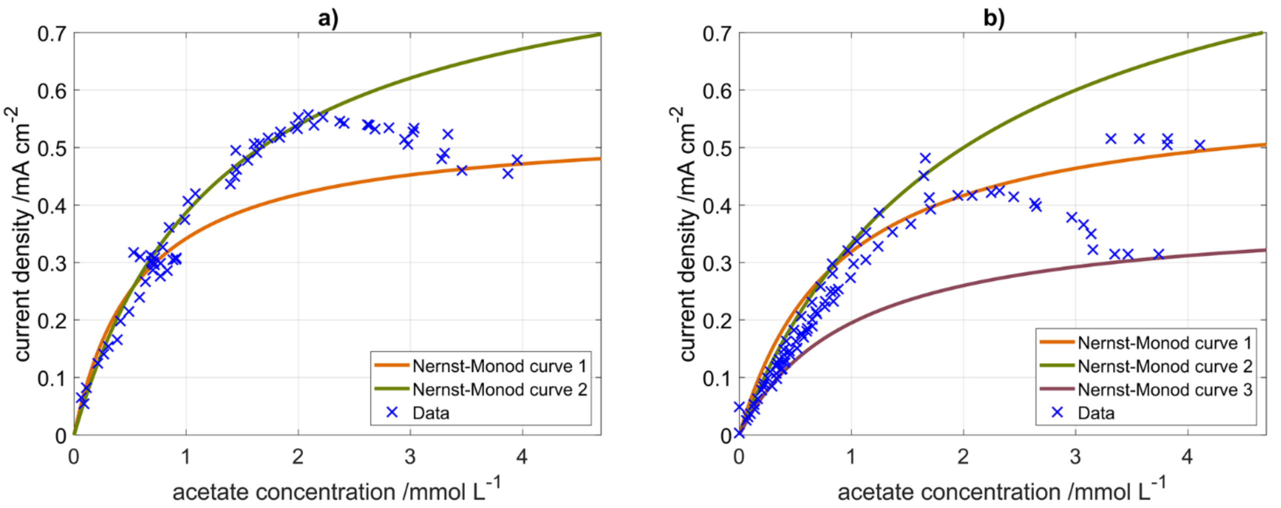

Figure S2: Nernst-Monod plot for double acetate pulses with a) continuous acetate supply between the two acetate pulses and b) a cell starvation phase without acetate supply between the two acetate pulses. The measured data points are displayed as markers, the solid lines indicate Nernst-Monod curves that were fitted to the data at different points in cultivation time. Nernst-Monod curves before (curve 1, orange) and after (curve 2, green) the acetate pulses as well as after the starvation phase (curve 3, purple) are shown.

In **Figure S2** the Nernst-Monod curves from a repetition of the double pulse experiments are shown. The data agree well with those from **Figure 5** in the main text.
